# International stroke genetics consortium recommendations for studies of genetics of stroke outcome and recovery

**DOI:** 10.1177/17474930211007288

**Published:** 2021-04-26

**Authors:** Arne G Lindgren, Robynne G Braun, Jennifer Juhl Majersik, Philip Clatworthy, Shraddha Mainali, Colin P Derdeyn, Jane Maguire, Christina Jern, Jonathan Rosand, John W Cole, Jin-Moo Lee, Pooja Khatri, Paul Nyquist, Stéphanie Debette, Loo Keat Wei, Tatjana Rundek, Dana Leifer, Vincent Thijs, Robin Lemmens, Laura Heitsch, Kameshwar Prasad, Jordi Jimenez Conde, Martin Dichgans, Natalia S Rost, Steven C Cramer, Julie Bernhardt, Bradford B Worrall, Israel Fernandez-Cadenas, International Stroke Genetics Consortium

**Affiliations:** 1 Department of Clinical Sciences Lund, Neurology, 5193 -->Lund University, Lund, Sweden; 2 Department of Neurology, Skåne University Hospital, Lund, Sweden; 3 Department of Neurology, University of Maryland, Baltimore, MD, USA; 4 Department of Neurology, University of Utah, Salt Lake City, UT, USA; 5 Department of Neurology, North Bristol NHS Trust, Bristol, UK; 6 Department of Neurology, 2647 -->The Ohio State University, Columbus, OH, USA; 7 Department of Radiology, University of Iowa, Iowa City, IA, USA; 8 Faculty of Health, University of Technology Sydney, Ultimo, NSW, Australia; 9 Department of Laboratory Medicine, Institute of Biomedicine, University of Gothenburg, Gothenburg, Sweden; 10 Department of Clinical Genetics and Genomics, Sahlgrenska University Hospital, Gothenburg, Sweden; 11 Department of Neurology, Massachusetts General Hospital, Boston, MA, USA; 12 Neurology Service, Baltimore Veterans Affairs Medical Center, Baltimore, MD, USA; 13 Department of Neurology, 12264 -->University of Maryland School of Medicine, Baltimore, MD, USA; 14 Department of Neurology, Washington University School of Medicine, St. Louis, MO, USA; 15 Department of Neurology and Rehabilitation Sciences, University of Cincinnati, Cincinnati, OH, USA; 16 Neurology, Anesthesiology/Critical Care Medicine, Neurosurgery, and General Internal Medicine, 1500 -->Johns Hopkins School of Medicine, Baltimore, MD, USA; 17 Bordeaux Population Health, Inserm U1219, University of Bordeaux, Bordeaux, France; 18 Neurology Department, Bordeaux University Hospital, Bordeaux, France; 19 Department of Biological Science, Faculty of Science, Universiti Tunku Abdul Rahman, Perak, Malaysia; 20 Department of Neurology, 12235 -->University of Miami Miller School of Medicine, Miami, FL, USA; 21 Department of Neurology, Weill Cornell Medicine, New York, NY, USA; 22 Stroke Theme, Florey Institute of Neuroscience and Mental Health, Melbourne, Vic, Australia; 23 Department of Neuroscience, University of Leuven, Leuven, Belgium; 24 Department of Neurology, University Hospitals Leuven, Leuven, Belgium; 25 Rajendra Institute of Medical Sciences, Ranchi, Jharkhand, India; 26 Neurology Department, Neurovascular Research Group, Institut Hospital del Mar d’Investigació Mèdica, Barcelona, Spain; 27 Neurology, Universitat Autònoma de Barcelona, Barcelona, Spain; 28 Institute for Stroke and Dementia Research, University Hospital, LMU, Munich, Germany; 29 Department of Neurology, UCLA, Los Angeles, CA, USA; 30 California Rehabilitation Institute, Los Angeles, CA, USA; 31 Department of Neurology, University of Virginia, Charlottesville, VA, USA; 32 Stroke Pharmacogenomics and Genetics Group, Sant Pau Biomedical Research Institute, Barcelona, Spain

**Keywords:** Data collection, genetics, ischemic stroke, outcome, phenotype, recovery, standardization

## Abstract

Numerous biological mechanisms contribute to outcome after stroke, including brain injury, inflammation, and repair mechanisms. Clinical genetic studies have the potential to discover biological mechanisms affecting stroke recovery in humans and identify intervention targets. Large sample sizes are needed to detect commonly occurring genetic variations related to stroke brain injury and recovery. However, this usually requires combining data from multiple studies where consistent terminology, methodology, and data collection timelines are essential. Our group of expert stroke and rehabilitation clinicians and researchers with knowledge in genetics of stroke recovery here present recommendations for harmonizing phenotype data with focus on measures suitable for multicenter genetic studies of ischemic stroke brain injury and recovery. Our recommendations have been endorsed by the International Stroke Genetics Consortium.

## Introduction

Genetic studies can potentially discover biological mechanisms affecting stroke recovery with treatment implications. However, they need large sample sizes only achievable by combining data from multiple studies, where harmonized terminology, methodology, and data collection timelines are essential.

The terms *stroke outcome* and *stroke recovery* differ in meaning. Stroke *outcome* describes the degree of function at specific time points; stroke *recovery* encompasses the degree of improvement (or deterioration) over time and better captures dynamic biological processes. Stroke recovery evaluation requires initial stroke severity data, without which only stroke outcome is measurable. It is also important to distinguish restitution (“true”) recovery from behavioral compensation. For example, “true” motor recovery suggests restoration of pre-stroke movement patterns^1^ whereas “compensation,” implies new (possibly dysfunctional) movement patterns for accomplishing functional tasks.^2^

The dynamics of stroke recovery depend on intrinsic and extrinsic factors.^3^ Each patient’s recovery pattern uniquely reflects the combined influences of lesion size and location, biological mechanisms of brain repair, comorbidities, pre-morbid health status, and post-stroke factors including acute recanalization, rehabilitation, psychosocial factors, and environmental influences. Consequently, the degree of stroke recovery varies considerably between individuals, and even skilled clinicians have difficulty making accurate recovery predictions.^4^

The need for improved predictive models of stroke recovery has become a major research focus^5,6^ and recent studies suggest that genetic variations influence recovery after stroke.^7–9^ Despite multiple studies, findings remain heterogeneous, due to differences in populations, recovery metrics, assessment time points, and study designs. Most studies using global assessments incorporate the modified Rankin Scale (mRS)^10^ while some use more detailed modality-specific functions, for example, upper extremity (UE) motor function, language or cognitive function,^3,11^ or patient-reported outcome measures (PROMs). Few studies use repeated measures, leading to knowledge gaps on stroke recovery time course. To standardize timing and metric choices across studies, the Stroke Recovery and Rehabilitation Roundtable taskforce in 2017 recommended core outcomes for trials and standardized measurement time points to reduce heterogeneity.^11^

Here, we focus specifically on design of prospective genetic studies of ischemic stroke (IS) recovery, aiming to ascertain the underlying genetic influences on stroke recovery biology. Our recommendations complement existing advise for standardizing phenotype data^12^ and biological sample collection^13^ for stroke risk and recovery studies^11,14^ by providing recommendations for pre-specified harmonized data sets suitable for large, high-quality, multi-center collaborations in prospective stroke genetic recovery studies. We propose measures comprehensive enough to provide both stroke- and domain-specific data, but simple enough to allow collection of large sample sizes across numerous and diverse enrollment sites. This will allow opportunities to discover genetic factors influencing hitherto unknown biological pathways affecting the dynamics of IS recovery. We do not here consider intracerebral hemorrhage (ICH) given ICH recovery mechanisms differ from IS.

## Methods

Methods for reaching a consensus on these recommendations are described in the Supplement.

## Results

### Overview of phenotypic variables

We prioritized phenotypic variables into categories: (1) *minimum variables*—mandatory for all studies, (2) *preferred variables—* recommended but may be precluded by practical limitations, and (3) *optional variables*—interesting for some multi-center projects. Table 1 shows the minimum (mandatory) variable set. Supplemental Table 1 lists a detailed comprehensive set. Supplemental Table 2 suggests variable formats to facilitate compilation of joint data. Regularly updated versions will be kept at the Global Alliance for International Stroke Genetics Consortium (ISGC) Acute and Long-term Outcome studies (https://genestroke.wixsite.com/alliesinstroke).

**Table 1.** Recommended *minimum* variable sets for genetic studies of ischemic stroke recovery.

Evaluation time	Clinical	Stroke clinical	Stroke imaging	Treatment	Functional
Stroke onset- two days	Pre-stroke/demographics^a^ • Pre-stroke mRS • Charlson Comorbidity Index^EP^ • Age at time of stroke onset • Sex • Race Risk factors at stroke onset^a^ • Hypertension • Atrial fibrillation • Coronary heart disease • Diabetes mellitus • Smoking • Hypercholesterolemia • Previous stroke	• Main stroke type^b^ • TOAST/CCS subtype • Survival^LP^ • Time from stroke to death^LP^	• Initial CT/MRI examination performed • Time to initial CT/MRI scan^LO^ • CT at 24 h^LO^ • Time to CT 24 h scan^LO^ • Hemorrhagic transformation on 24 h CT scan^LO^	• Thrombolysis • Thrombectomy	• Initial stroke severity: NIHSS^c^ within 6 h^d^ after hospital presentation (when possible) or just before recanalization therapy • Time from stroke onset to initial NIHSS^LP^ • NIHSS^c^ 24 h after recanalization therapy/24 h after baseline NIHSS, if no recanalization therapy^LO^ • Time from stroke onset to 24 h NIHSS^LO^
Seven days/ discharge	As above, if not already done	• Survival • Time from stroke to death^LP^			• NIHSS^c^ at seven days or at discharge, if earlier^EP^ • Time from stroke onset to NIHSS at seven days/discharge^EP^
Three months	• Time of evaluation^EO^ • Survival^EO^ • Recurrent stroke^EO^				• NIHSS^c,EO^ • mRS^EO^
12 months, yearly thereafter	• Time of evaluation^EO^ • Survival^EO^ • Recurrent stroke^EO^				• NIHSS^c,EO^ • mRS^EO^

Listed variables are recommended as *minimum* for Early Phase Studies (with focus on 0–48 h and seven days/hospital discharge) and Later Phase Studies (with focus on three months and beyond), unless otherwise specified. A comprehensive outline of all suggested *minimum*, *preferred*, and *optional* variables are shown in Supplemental Table 1. Time of evaluation after stroke onset (hours for up to 72 h; days thereafter) should be registered.

mRS: modified Rankin Scale; IS: ischemic stroke; ICH: intracerebral haemorrhage; h: hours; NIHSS: National Institutes of Health Stroke Scale; CT: computed tomography; MRI: magnetic resonance imaging; TOAST: Trial of ORG 10172 in Acute Stroke Treatment; CCS: Causative Classification of Stroke.

a Can often be collected somewhat later.

b Only IS in this manuscript.

c Including individual subitems.

d For Later Phase studies: NIHSS within 6 h = *preferred*, NIHSS within 24 h = *minimum*.

EP Early Phase studies, *preferred*.

LP Later Phase studies, *preferred.*

EO Early Phase studies, *optional*.

LO Later Phase studies, *optional*.

### Timing of recovery assessment

**Figure 1.**
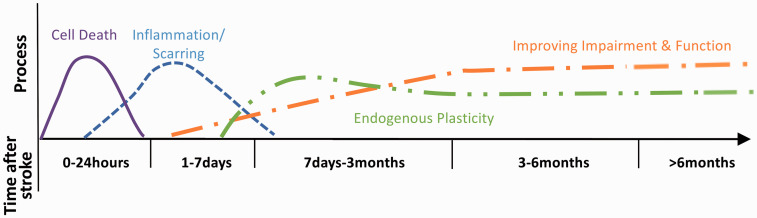
Framework showing time points post stroke related to current known biology of stroke recovery. Time post stroke should always be included in data acquisition. Adapted to represent ischaemic stroke only, from: Bernhardt J et al, Int J Stroke 2017, Vol. 12(5) 444–450, copyright © 2017 by World Stroke Organization. Reprinted by permission of SAGE Publications, Ltd.

Stroke recovery starts immediately at symptom onset and continues for years thereafter (Figure 1). Blood biomarkers, and other biomarker evaluations, for example, magnetic resonance imaging (MRI), often vary across time points. To provide simplification, we recommend the time course for assessment of evolution and recovery into three phases post-stroke (Day 0 is day of stroke onset): 0 to 24–48 h, seven days, and approximately Day 90 after stroke onset and when possible one year and later. When appropriate, studies may choose additional precisely defined time periods.

Studies of hyperacute recovery and therapy should perform evaluations within 6 h (when possible) or at least within 24 h after stroke onset and before revascularization therapy, followed by a new evaluation at 24 h post stroke^15^ or 24 h after recanalization therapy (see below).

Seven days post-stroke is often recommended for evaluation.^1^ However, because many patients leave hospital earlier, we recommend evaluation either at seven days or discharge, whichever occurs earlier.

IS studies often conclude evaluations at three months.^16,17^ However, improvement may occur at 6–12 months and possibly beyond.^18^ Recovery is not linear, and time frames may vary by different domains, for example, cognitive versus motor function.^19^ To evaluate three-month recovery independently of early acute phases, sometimes influenced by treatments, for example, revascularization, we recommend measuring recovery as functional change between Day 7 (or discharge), and three months. If possible, additional evaluations at one and three years are strongly recommended to evaluate longer term recovery.

### Recommended phenotypic variables

#### Pre-stroke variables, demographic data

Pre-stroke functional status affects stroke outcome and should be measured as mRS, ideally specifying whether due to a stroke preceding the index stroke versus other conditions. We also recommend the Charlson Comorbidity Index,^20^ with information about pre-existing key medical conditions. For further details, see Table 1 and Supplemental Table 1.

All studies should provide demographics: age at stroke onset, sex, race/ethnicity, residential area type (urban/rural), educational status, living situation (housing type), and available social support (living alone/with someone).^21^

#### Baseline clinical and imaging information

Baseline characteristics of current IS should include initial National Institutes of Health Stroke Scale (NIHSS) total and individual component scores, and Trial of ORG 10172 in Acute Stroke Treatment^22^ and/or Causative Classification of Stroke subtype.^23^ Specific “other determined” stroke etiologies (e.g., cervical artery dissection) could be detailed. Laboratory parameters and Glasgow Coma Scale may be recorded.

We recommend baseline registration of head computed tomography/magnetic resonance (CT/MRI), and CT/MRI angiography and perfusion, because, for example, dynamic blood flow changes may be related to genetic influences.^24^

#### Stroke treatment and neuroimaging at 0–48 h and seven days/hospital discharge

Treatment with thrombolysis and thrombectomy should be noted. Final expanded thrombolysis in cerebral infarction (TICI) score^25^ indicating degree of revascularization achieved should be mandated in large vessel occlusion stroke studies. Additional treatments possibly affecting recovery include carotid endarterectomy/stenting and pharmacologic interventions for blood pressure, dyslipidemia, or atrial fibrillation.

Follow-up imaging at 24 h after recanalization therapy with CT/MRI is valuable to evaluate location and extent of the acute ischemic lesion(s). When possible, MRI with FLAIR, DWI, MRI angiography, and GRE/T2*/SWI is recommended within 24 h (or within seven days at the latest) after stroke onset. MRI performed later might also have value. Imaging measures of leukoaraiosis, microhemorrhages, prior infarcts, and arterial stenoses could be considered. Injury extent to specific neural structures, such as corticospinal tract, may be useful for some hypotheses.

Neuroimaging biomarkers can serve as endophenotypes. For examples, please see Supplement.

#### Clinical measures at 0–48 h and seven days/hospital discharge

In the first days after stroke, neurological deficits can be highly unstable, with patients rapidly improving, or deteriorating. Serial NIHSS scores,^26^ often standard of care in acute stroke, capture these changes. A change in NIHSS between baseline (<6 h from onset) and 24 h (ΔNIHSS_6–24 h_) is related to 90-day outcome independent of baseline NIHSS^27^ with a genome wide association study (GWAS) of ΔNIHSS_6–24 h_ having revealed genes potentially involved in ischemic brain injury (data not shown). We therefore recommend NIHSS (including subitems) at baseline <6 h or at least within 24 h after stroke onset, and short-term follow-up at 24 h after stroke onset/after recanalization therapy, noting number of hours since stroke onset.

Recovery during the initial days after stroke onset is difficult to measure, and we recommend evaluations including NIHSS (with subitems) either at seven days or discharge from hospital, whichever occurs earlier.

The Shoulder Abduction Finger Extension score during the first three days after stroke predicts upper limb motor outcome.^28^ This complements the NIHSS and is useful as an early marker, easier to assess than more complex motor assessments such as the Fugl-Meyer (FM) or Action Research Arm Test (ARAT).

Gait performance measured as walking speed predicts walking recovery and falls risk. Gait is also linked with quality of life and participation level, and testing does not require much time. On Day 7, we recommend recording the ability to walk 10 m independently (yes/no), and for those able, a 10 -m walk test. This may be repeated at later time points (see below).

Early complications such as infections and recurrent stroke may also influence recovery and should be considered.

#### Considerations and treatment information up to three months and beyond

Stroke recurrence, with a 30%–40% cumulative risk among first stroke survivors, is a common cause of worsening disability and requires tracking.^29,30^ Secondary prevention measures, complications (e.g., depression, infections, seizures, fractures after falls), level of physical activity, and socioeconomic factors may substantially affect outcome and recovery. At the designing stage, studies should define which of these variables to collect as confounding factors, exclusion criteria, or endpoint/dependent variables.

Rehabilitation treatment is heterogeneous across centers and difficult to uniformly register. We suggest registering how often the treatment is administered per week or month and duration of rehabilitation in days. The starting day after stroke onset and treatment dose (minutes per day) may be recorded.

Treatment with antidepressants and other psychotropic medication^31^ should be noted as should other rehabilitation adjuncts, whether pharmacologic or device-based (e.g., transcranial magnetic stimulation).

#### Evaluation at three months and beyond

Factors influencing long-term recovery (improvement/deterioration) may differ from those important in earlier time periods. As mentioned above, we recommend evaluation at Day 7, or discharge (if earlier) as a new baseline for long-term recovery at three months.

**Figure 2.**
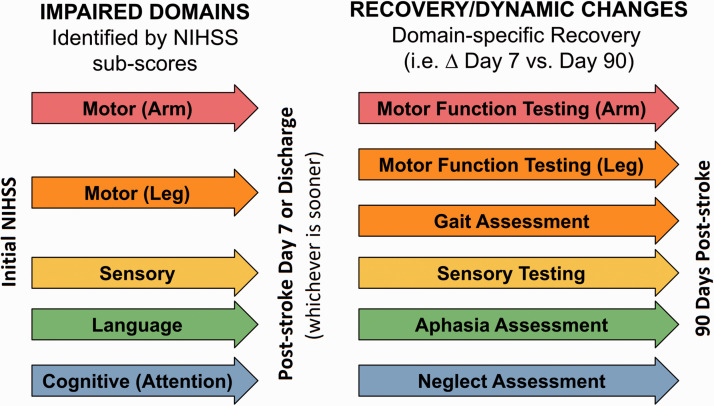
Suggested domain-specific screening by using NIHSS. Detected deficits are subsequently assessed with more detailed evaluations. NIHSS: National Institutes of Health Stroke Scale.

Stroke variably affects different functional domains.^32^ We recommend that specific domains are considered separately and only in more detail where appropriate. For example, if a motor deficit is detected on the NIHSS, more in-depth motor testing can be performed (Figure 2 and Supplemental Table 1). In this way, the NIHSS subitems provide screening for deficits requiring more detailed evaluation, saving time, and resources.

### Evaluation of specific recovery domains

#### Motor function

Motor deficits are seen in >80% of IS^33^ and can be screened by NIHSS items 5 and 6. A more detailed assessment of motor deficit changes over time is of great importance to evaluate recovery. The FM-UE motor scale^34^ is well known and recommended but requires trained personnel.^35^ The FM lower extremity motor scale may be considered,^34^ but limited reproducibility, a high concordance with UE weakness, and overlapping recovery mechanisms may limit its value. UE motor function is best captured with ARAT, but this requires equipment.^11^

Gait velocity (see above) is also useful for long-term motor function evaluation.

#### Sensory function

The FM Sensory Examination or the Nottingham Sensory Scale could be considered.

#### Cognitive function

Combining the four NIHSS items, Orientation (1b), Executive function (1c), Language (9), and Inattention (11), has similar value as the Mini-Mental State Examination in detecting severe cognitive impairment.^36^ A more elaborate cognitive evaluation with the Montreal Cognitive Assessment Scale^37^ is recommended when possible. When even more detailed or longitudinal understanding of specific cognitive domains is needed, an in-depth neuropsychological assessment may encompass multiple cognitive domains, especially verbal episodic memory, executive function, and processing speed. Pre-stroke cognitive assessment with tools such as the informant questionnaire on cognitive decline in the elderly (IQCODE)^38^ is important, as pre-stroke cognitive impairment is frequent and associated with post-stroke dementia.^39^ The genetics of post-stroke cognitive impairment are not covered here but addressed in separate working groups of the ISGC (www.strokegenetics.org) and the Cohorts for Heart and Aging Research in Genomic Epidemiology consortium.

#### Speech function

NIHSS item 9 provides a screening tool for aphasia. Aphasia evaluations are hampered by language differences between populations. We favor the Western Aphasia Battery-Revised version bedside screening test, which takes 10–15 min and is well-accepted by researchers.^40^ Language evaluation items in cognitive tests are also a possibility.

#### Neglect

NIHSS item 11 provides a screening tool for neglect and hemi-inattention. Among the many available bedside assessments, the Star cancellation test is recommended.

#### Mood

The Hospital Anxiety and Depression Scale^41^ had most consensus in our group for utility across different time points. Alternatives have been recommended by others.^42^

#### Other specific domains

Post-stroke visual field loss, eye movement abnormalities, dysphagia, balance disorders, fatigue, frailty, and urinary incontinence are all important aspects of post-stroke recovery. We agreed that no specific recommendations can be made for these domains at this time but provide some suggestions in the Supplement.

#### Global assessment

The three-month mRS is used in most stroke trials and should be performed in studies of stroke recovery genetics to facilitate comparison across cohorts. Evaluation of mRS at other time points (e.g., 6 months, 12 months, and yearly thereafter) may be useful. The mRS offers advantages of ease of administration, good inter-observer reproducibility, certification, and available phone-based evaluation.^10,43^. The mRS score has been analyzed both as a continuous and an ordinal variable,^44,45^ but dichotomization may lose information and statistical power.

Other functional scales, such as Barthel Index and the Nottingham extended activities of daily living scale (ADL), have limitations such as ceiling effects or rarer usage.

#### Patient-reported outcome measures

Outcome and recovery evaluations important to clinicians are not always congruent with those of patients. When possible, PROMs should be included in recovery studies to support the validity of other measures and reflect meaningful stroke outcomes and recovery. PROMs can assess disability, mood, cognitive function, pain, mobility, and fatigue. The Patient-Reported Outcome Measurement Information System, 36-Item Short Form Survey, EuroQuality of life 5 dimension questionnaire (EQ-5D), and Stroke Impact Scale are frequently used PROMs.^46^

#### Combining dynamic changes from different domains

Genetic correlates of recovery mechanisms may influence several functional domains. Combined measurements across domains can be obtained by quantification of the domain with greatest impairment in individual subjects (defined as the system with the worst baseline subscore from the baseline NIHSS), and computing the percentage of the maximum possible score for this domain followed by comparing these measures on Day 7 and Day 90. Recovery is calculated as the remaining deficit (% recovery = 100 × (1−(ScoreMax − Score_d90_)/(ScoreMax − Score_d7_))) for each subject.^47^

#### Neuroimaging

Neuroimaging after stroke can detect new infarcts, hemorrhages, and small vessel disease including white matter changes and brain atrophy. For these purposes, MRI including FLAIR and GRE/T2*/SWI sequences could be considered at three months, one year, and later.

Several other forms of neuroimaging and associated methods have been examined in relation to genetic variation, for examples—please see Supplement.

## Discussion

We here recommend a specific set of phenotype outcome variables, time frames, and covariates for prospective genetic studies of recovery after IS. To detect changes in the patient-specific evolution of symptoms the same variables should, when possible, be measured at the different time points.

Our suggested time points for evaluations and the assessments categorized as minimum, preferred, or optional can be useful tools for individual studies, comparative studies, and multi-center studies on stroke recovery genetics. Of the large number of potential evaluation tools available for assessment of IS recovery, we suggested tools that should be simple and accessible while detailed enough to capture dynamic changes in the designated domains.

Physical follow-up examinations after the acute phase of stroke are labour intensive. Patient telephone interviews may be an alternative. Live examinations permit detailed determination of many neurological features but come at a higher price such as cost and travel. Phone and video-based examinations are less expensive, but more limited in the data that can be reliably measured. Given the focus of the current recommendations, we advise live examinations for studies focusing on recovery at 90 days and beyond to be performed whenever resources permit.

We stress the use of NIHSS, including its subscores, for screening because it is already widely utilized in clinical routine, clinical trials, and recovery studies. More elaborate evaluations focusing on specific domains can be complementary, as can combined measures such as the predict recovery potential 2 algorithm evaluating clinical function, MRI, and surrogate parameters to predict three-month UE motor function.^28^ Other clinical evaluations to predict recovery such as sitting balance for independent walking and ability to comprehend and repeat spoken language are uncommonly standardized and systematically investigated and may currently have less value for genetic studies of stroke recovery. Increasing importance is being placed on PROMs to ensure that recovery measured using tools based on neurological impairment is meaningful from the patient’s perspective, although the role of PROMs in stroke genetics research has not been established.

Training, certification, and recertification are essential to reduce error and inter-rater variance. A plan for training, certification, and recertification for each behavioral scale should be a part of every stroke recovery study or trial.

Statistical considerations are important. Many scales are ordinal and non-linear. An improvement in the NIHSS of 10 points, for instance, may signify different degrees of improvement when a patient improves from 20 to 10 versus from 10 to 0. Additional concerns regarding repeated measurements include regression to the mean and management of missing data. Analyses must consider collinearity when employing the same variable to calculate both the independent and the dependent variables to avoid misinterpretation of paired observations when comparing baseline scores with follow-up results.^48^ Analyses combining different domains may be considered for detecting genetic influence on general stroke recovery.

## Conclusions

The rapid progress of genetic research methodologies provides excellent opportunities to discover new factors influencing stroke recovery. However, to obtain optimal efficiency, harmonized and well-accepted phenotyping instruments across studies are required. We suggest selected evaluations of stroke recovery to measure important recovery domains. Harmonization of these evaluations between studies will allow performance of large prospective studies of genetic influence on recovery dynamics in the early and later phases after stroke.

## Supplemental Material

sj-pdf-1-wso-10.1177_17474930211007288 - Supplemental material for International stroke genetics consortium recommendations for studies of genetics of stroke outcome and recovery Click here for additional data file. Supplemental material, sj-pdf-1-wso-10.1177_17474930211007288 for International stroke genetics consortium recommendations for studies of genetics of stroke outcome and recovery by Arne G Lindgren, Robynne G Braun, Jennifer Juhl Majersik, Philip Clatworthy, Shraddha Mainali, Colin P Derdeyn, Jane Maguire, Christina Jern, Jonathan Rosand, John W Cole, Jin-Moo Lee, Pooja Khatri, Paul Nyquist, Stéphanie Debette, Loo Keat Wei, Tatjana Rundek, Dana Leifer, Vincent Thijs, Robin Lemmens, Laura Heitsch, Kameshwar Prasad, Jordi Jimenez Conde, Martin Dichgans, Natalia S Rost, Steven C Cramer, Julie Bernhardt, Bradford B Worrall, Israel Fernandez-Cadenas and International Stroke Genetics Consortium in International Journal of Stroke

sj-pdf-2-wso-10.1177_17474930211007288 - Supplemental material for International stroke genetics consortium recommendations for studies of genetics of stroke outcome and recovery Click here for additional data file. Supplemental material, sj-pdf-2-wso-10.1177_17474930211007288 for International stroke genetics consortium recommendations for studies of genetics of stroke outcome and recovery by Arne G Lindgren, Robynne G Braun, Jennifer Juhl Majersik, Philip Clatworthy, Shraddha Mainali, Colin P Derdeyn, Jane Maguire, Christina Jern, Jonathan Rosand, John W Cole, Jin-Moo Lee, Pooja Khatri, Paul Nyquist, Stéphanie Debette, Loo Keat Wei, Tatjana Rundek, Dana Leifer, Vincent Thijs, Robin Lemmens, Laura Heitsch, Kameshwar Prasad, Jordi Jimenez Conde, Martin Dichgans, Natalia S Rost, Steven C Cramer, Julie Bernhardt, Bradford B Worrall, Israel Fernandez-Cadenas and International Stroke Genetics Consortium in International Journal of Stroke

sj-pdf-3-wso-10.1177_17474930211007288 - Supplemental material for International stroke genetics consortium recommendations for studies of genetics of stroke outcome and recovery Click here for additional data file. Supplemental material, sj-pdf-3-wso-10.1177_17474930211007288 for International stroke genetics consortium recommendations for studies of genetics of stroke outcome and recovery by Arne G Lindgren, Robynne G Braun, Jennifer Juhl Majersik, Philip Clatworthy, Shraddha Mainali, Colin P Derdeyn, Jane Maguire, Christina Jern, Jonathan Rosand, John W Cole, Jin-Moo Lee, Pooja Khatri, Paul Nyquist, Stéphanie Debette, Loo Keat Wei, Tatjana Rundek, Dana Leifer, Vincent Thijs, Robin Lemmens, Laura Heitsch, Kameshwar Prasad, Jordi Jimenez Conde, Martin Dichgans, Natalia S Rost, Steven C Cramer, Julie Bernhardt, Bradford B Worrall, Israel Fernandez-Cadenas and International Stroke Genetics Consortium in International Journal of Stroke
