# Supplementary material for: International stroke genetics consortium recommendations for studies of genetics of stroke outcome and recovery
Source: Int J Stroke. 2021 Apr 26;17(3):260–8. doi: 10.1177/17474930211007288 (PMC8864333; doi:10.1177/17474930211007288)
Supplement: sj-pdf-1-wso-10.1177_17474930211007288 - Supplemental material for International stroke genetics consortium recommendations for studies of genetics of stroke outcome and recovery [file sj-pdf-1-wso-10.1177_17474930211007288.pdf]

Supplemental Material

International stroke genetics consortium recommendations for studies of genetics of stroke outcome and recovery

Arne G Lindgren, Robynne G Braun, Jennifer Juhl Majersik, Philip Clatworthy, Shraddha Mainali, Colin P Derdeyn, Jane Maguire, Christina Jern, Jonathan Rosand, John W Cole, Jin-Moo Lee, Pooja Khatri, Paul Nyquist, Stéphanie Debette, Loo Keat Wei, Tatjana Rundek, Dana Leifer, Vincent Thijs, Robin Lemmens, Laura Heitsch, Kameshwar Prasad, Jordi Jimenez Conde, Martin Dichgans, Natalia S Rost, Steven C Cramer, Julie Bernhardt, Bradford B Worrall and Israel Fernandez-Cadenas; International Stroke Genetics Consortium

1. Methods……………………………………………………………….…………..….page S1
2. Examples of imaging and other methods that have been examined in relation

to genetic variation and stroke outcome, including references………………….……...page S2

3. Acknowledgements……………………………………………………..……....page S3

4. Disclosures……………………………………………………………………...…page S4

Methods

The authors of this manuscript are stroke and rehabilitation clinicians and researchers with knowledge in genetics of stroke recovery. They were identified and contacted through the International Stroke Genetics Consortium (ISGC) networks, working groups and initiatives focusing on stroke recovery including Genetics of Ischaemic Stroke Functional Outcome (GISCOME), Global Alliance for ISGC Acute and Long-term Outcome studies, Genomic Platform for Acute Stroke Drug Discovery (GPAS); and Stroke Recovery and Rehabilitation Roundtable taskforce (SRRR).

A formal Delphi process for reaching consensus was not used. Instead, an agreement on the recommendations presented was obtained after extensive in-person meetings, telephone conferences and e-mail correspondence between 2017 and 2020. During a first round of meetings and correspondence, the group compiled an over-inclusive list of potential outcome measures. These measures were separately prioritised by each group member before a further round of meetings, including at ISGC workshops, and correspondence led to a consensus regarding which measures would be included in the recommendations and which would be considered essential (i.e. part of the minimum variable set), preferred, or optional. The final recommendations were subsequently endorsed by the ISGC.

Examples of imaging and other methods that have been examined in relation to genetic variation and stroke outcome.

- In the acute phase hemorrhagic transformation can be described as either categorical variables (HT1, HT2, PH1, or PH2) or a continuous variable (hemorrhage volume).[1] Likewise, serial CT scans can quantify cerebral edema formation as change in CSF volume over time,[2] or change in lesion water uptake.[3] Automated methods could assess thousands of images required for GWAS.[4]

- At follow-up, examples of methods examined in relation to genetic variation and stroke outcome include structural imaging,[5] functional imaging[6] (e.g., task-related activation and connectivity) (including T1, DTI and fMRI),[7] and electrophysiological assessment (e.g., Transcranial Magnetic Stimulation, EEG, MEG).[8]

References

1. Torres-Aguila NP, Carrera C, Muino E, et al. Clinical variables and genetic risk factors associated with the acute outcome of ischemic stroke: A systematic review. *J Stroke*. 2019;21:276-289
2. Dhar R, Yuan K, Kulik T, et al. Csf volumetric analysis for quantification of cerebral edema after hemispheric infarction. *Neurocrit Care*. 2016;24:420-427
3. Broocks G, Flottmann F, Scheibel A, et al. Quantitative lesion water uptake in acute stroke computed tomography is a predictor of malignant infarction. *Stroke*. 2018;49:1906-1912
4. Dhar R, Chen Y, An H, et al. Application of machine learning to automated analysis of cerebral edema in large cohorts of ischemic stroke patients. *Front Neurol*. 2018;9:687
5. Rutten-Jacobs LCA, Tozer DJ, Duering M, et al. Genetic study of white matter integrity in uk biobank (n=8448) and the overlap with stroke, depression, and dementia. *Stroke*. 2018;49:1340-1347
6. Kim DY, Quinlan EB, Gramer R, et al. Bdnf val66met polymorphism is related to motor system function after stroke. *Phys Ther*. 2016;96:533-539
7. Liew SL, Zavaliangos-Petropulu A, Jahanshad N, et al. The enigma stroke recovery working group: Big data neuroimaging to study brain-behavior relationships after stroke. *Hum Brain Mapp*. 2020
8. Burke E, Cramer SC. Biomarkers and predictors of restorative therapy effects after stroke. *Curr Neurol Neurosci Rep*. 2013;13:329

Acknowledgements:

Dr Lindgren: The Swedish Research Council (2019-01757), CaNVAS project NIH (1R01NS114045-01), The Swedish Government (under the “Avtal om Läkarutbildning och Medicinsk Forskning, ALF”), The Swedish Heart and Lung Foundation, Region Skåne, Lund University, Skåne University Hospital, Sparbanksstiftelsen Färs och Frosta, Fremasons Lodge of Instruction Eos in Lund.

Dr. Braun: NIH/NICHD (K12HD093427).

Dr. Majersik: NIH/NINDS (5U10NS086606). Editorial Board for Stroke and Neurology. Consultant for Foldax (minor).

Dr. Jern: The Swedish Heart and Lung Foundation (20190203), the Swedish Research Council (2018-02543), the Swedish Government (under the “Avtal om Läkarutbildning och Medicinsk Forskning, ALF”) (ALFGBG-720081).

Dr. Cole: NIH (R01-NS114045; R01-NS100178; R01-NS105150), the US Department of Veterans Affairs, the AHA (15GPSPG23770000;17IBDG33700328).

Dr. Lee: NIH R01NS085419, U24NS107230.

Dr. Leifer: NIH U24-NS107237.

Dr. Lemmens: Senior Clinical Investigator of FWO Flanders (1841918N).

Dr. Heitsch: NIH-NINDS (K23NS099487-01).

Dr. Prasad: Department of Biotechnology, Govt. of India.

Dr. Rost: NIH-NINDS (R01-NS082285; R01-NS086905; U19-NS115388).

Dr. Worrall: R21-NS106480; U24-NS107222; KL2TR003016.

Dr. Fernandez-Cadenas: Maestro Project and Ibiostroke project funded by Eranet-Neuron, ISCIII and FEDER.

Disclosures:

Dr. Lindgren: Personal fees from Bayer, Astra Zeneca, BMS Pfizer, and Portola.

Dr. Majersik: Editorial Board Stroke and Neurology. Consultant for Foldax.

Dr. Rosand: Consulting fees from Boehringer Ingelheim; Editorial Boards, Lancet Neurology and European Stroke Journal.

Dr. Lee: Grant support from Biogen, Consultant for Regenera.

Dr. Lemmens: Institutional fees from Bayer, Boehringer Ingelheim, Genentech, Ischemiaview and Medtronic.

Dr. Cramer: Consultant for Abbvie, Constant Therapeutics, MicroTransponder, Neurolutions, Regenera, SanBio, Stemedica, Fujifilm Toyama Chemical Co., Biogen, and TRCare.

Dr. Bernhardt: Associate Editor, International Journal of stroke, Section Editor, Stroke.

Dr. Worrall: Deputy Editor for Neurology from the AAN.

The other authors report no disclosures.
