# Supplementary material for: International stroke genetics consortium recommendations for studies of genetics of stroke outcome and recovery
Source: Int J Stroke. 2021 Apr 26;17(3):260–8. doi: 10.1177/17474930211007288 (PMC8864333; doi:10.1177/17474930211007288)
Supplement: sj-pdf-2-wso-10.1177_17474930211007288 - Supplemental material for International stroke genetics consortium recommendations for studies of genetics of stroke outcome and recovery [file sj-pdf-2-wso-10.1177_17474930211007288.pdf]

Supplemental Table I.
Comprehensive table of recommended variables for prospective genetic ischemic stroke recovery studies.
The table provides an overview of variable prioritization. The three priority levels "minimum" (=mandatory), "preferred" (=recommended) and "optional" depend on whether a genetic study is primarily focusing on early recovery at 0-48 hours up to 7 days/discharge, or recovery at 90 days/3months and beyond, as outlined in the two columns to the right. Recommended formats for specific variables are in more detail described in Supplemental Table II.

**Domain**	**Method**	**Priority level**	
		Studies focusing on recovery at 0-48 hours up to 7 days/discharge from hospital	Studies focusing on recovery at 90 days/3 months and beyond
**1. Clinical Details: Pre-stroke and demographics (recommended to be assessed between day 0-7/discharge)**			
Pre-stroke functional status	Pre-stroke mRS	Minimum	Minimum
Comorbidity	Charlson comorbidity Index‡	Preferred	Minimum
Social support	Living alone or with someone	Optional	Preferred
Living situation	Housing type	Optional	Preferred
Residential area	Urban/Rural	Optional	Preferred
Educational status	Years of education	Optional	Preferred
Age	Age at time of stroke	Minimum	Minimum
Sex	Sex	Minimum	Minimum
Race	Race	Minimum	Minimum
Physical activity	†	Preferred	Optional
Cognition	IQCODE	Optional	Preferred
Handedness	Dominant hand	Optional	Preferred
**Medication ongoing at stroke onset**			
Medication	†	Optional	Optional
Ongoing* anti-platelet treatment	Yes/No	Preferred	Preferred
Ongoing* anticoagulation treatment	Yes/No	Preferred	Preferred
Ongoing* anti-depressant treatment	Yes/No	Preferred	Preferred
**Cardiovascular risk factors at stroke onset**			
Hypertension	Yes/No	Minimum	Minimum
Atrial fibrillation	Yes/No	Minimum	Minimum
Coronary Heart Disease	Yes/No	Minimum	Minimum
Diabetes Mellitus**	Yes/No	Minimum	Minimum
Smoking	Current/Former/Never	Minimum	Minimum
Hypercholesterolemia	Yes/No	Minimum	Minimum
Previous stroke	Yes/No	Minimum	Minimum

Type of previous stroke	IS/ICH	Preferred	Preferred
Previous TIA	Yes/No	Preferred	Preferred
**2. Stroke and imaging details up to 7 days after stroke onset**			
**Stroke clinical details**			
Main stroke type (IS, ICH)	Only IS included in this manuscript	Minimum	Minimum
Ischemic stroke subtype according to TOAST[22] or CCS[23]	†	Minimum	Minimum
TOAST/CCS details on "other determined" causes of stroke	†	Preferred	Preferred
BP at hospital presentation	†	Preferred	Optional
Glucose at hospital presentation	†	Preferred	Optional
White blood cell count	†	Preferred	Preferred
Blood platelet count	†	Optional	Optional
Infection post stroke	Yes/No	Preferred	Preferred
Survival	Yes/No	Minimum	Minimum
Time from stroke onset to death	Hours/Days †	Minimum	Preferred
**Stroke imaging details**			
CT/MR	CT/MR/both/none performed	Minimum	Minimum
Initial CTA	CTA examination performed Yes/No	Preferred	Preferred
Initial CTP	CTP examination performed Yes/No	Preferred	Preferred
Time to initial CT/MR scan	Hours	Minimum	Optional
Lesion location on CT/MR	†	Preferred	Preferred
Lesion volume on CT/MR	mL	Preferred	Preferred
Type of MR sequences	†	Optional	Optional
Arterial stenosis on DSA/CTA/MRA in relevant vessel	Yes/No	Optional	Optional
Collateral blood flow on DSA/CTA/MRA	†	Optional	Optional
CT 24h	Yes/No	Minimum	Optional
CTA 24h	Yes/No	Preferred	Optional
Time to CT 24h scan	Hours	Minimum	Optional
MRI 24h (or within 3 days at the latest)	(Yes/No)<br>Size of core on DWI (mL), CTP, cortical spinal tract involvement	Preferred	Optional
Time to MR 24h scan	Hours	Preferred	Optional

Type of MRI sequences	†	Optional	Optional
Lesion location on MRI	†	Preferred	Preferred
Lesion volume on MRI	mL	Preferred	Preferred
Microhemorrhages on MRI	Yes/No	Optional	Optional
Prior infarct on CT/MRI	Yes/No	Optional	Optional
Prior infarct modality	CT, MR, both, none	Optional	Optional
Leukoaraiosis on CT/MRI	Yes/No	Optional	Optional
Prior infarct modality	CT, MR, both, none	Optional	Optional
Hemorrhagic transformation (ECASS criteria)	†	Minimum	Optional
Edema	†	Preferred	Optional
**3. Acute stroke treatment up to 7 days from stroke onset**			
Thrombolysis	Thrombolysis treatment provided Yes/No	Minimum	Minimum
Agent used	Alteplase/Tenecteplase	Preferred	Optional
Time to thrombolysis	Minutes	Preferred	Optional
Thrombectomy	Thrombectomy treatment provided Yes/No	Minimum	Minimum
Time to thrombectomy	Minutes	Preferred	Optional
eTICI score after procedure	†	Preferred	Optional
Decompressive craniectomy	Yes/No	Optional	Optional
Cervical artery endarterectomy or stenting	Yes/No	Optional	Optional
BP treatment	Yes/No	Optional	Optional
**4. Functional scores up to day 7 from stroke onset**			
Initial stroke severity: NIHSS within 6h after hospital presentation (when possible) or just before recanalization therapy	NIHSS‡	Minimum	Preferred‖
Time from stroke onset to initial NIHSS	Hours	Minimum	Preferred
Physical barrier to speech at initial assessment	Yes/No	Preferred	Optional
Initial Glasgow Coma Scale	Initial GCS	Preferred	Optional
NIHSS 24h after recanalization therapy	NIHSS‡	Minimum	Optional

/24h after baseline NIHSS, if no recanalization therapy			
Time from stroke onset to 24h NIHSS	Hours	Minimum	Preferred
Physical barrier to speech at 24h assessment	Yes/No	Preferred	Optional
SAFE score within first 72 hours	†	Optional	Preferred
NIHSS at day 7 or at discharge if earlier	NIHSS‡	Preferred	Minimum
Time from stroke onset to 7 day/discharge NIHSS	Days	Preferred	Minimum
Physical barrier to speech at 7 day/discharge assessment	Yes/No	Preferred	Preferred
Fugl-Meyer UE motor at day 7 or at discharge if earlier	†	Optional	Preferred
Fugl-Meyer LE motor at day 7 or at discharge if earlier	†	Optional	Optional
Fugl-Meyer UE sensory at day 7 or at discharge if earlier	†	Optional	Optional
Fugl-Meyer LE sensory at day 7 or at discharge if earlier	†	Optional	Optional
ARAT	†	Optional	Optional
Independent walking	Ability to walk 10 m independently y/n	Preferred	Preferred
10 m walk test	Seconds	Preferred	Preferred
Cognitive status at 7 days or earlier at discharge	MoCA test‡	Optional	Preferred
**5. Considerations and treatment information at 90 days/3 months and beyond**			
Social support	Living alone or with someone	Optional	Preferred
Rehab treatment†	Yes/No	Optional	Preferred
Rehab treatment – start day after stroke onset	Start day after stroke onset	Optional	Optional
Rehab treatment – intensity	†	Optional	Preferred
Other Rehab adjunct	†	Optional	Preferred
Antidepression treatment†	Yes/No	Optional	Preferred
Secondary prevention	†	Optional	Preferred

6. Evaluation at 90 days/3 months and beyond			
**At 90 days**			
Time of evaluation for 90-day assessment, days since stroke	Days from stroke onset	Optional	Minimum
Survival	Yes/No	Optional	Minimum
Days from stroke onset to death	Days from stroke onset	Optional	Preferred
NIHSS	NIHSS‡	Optional	Minimum
mRS	mRS	Optional	Minimum
Barthel Index	BI‡	Optional	Optional
Fugl-Meyer UE motor	†	Optional	Preferred
Fugl-Meyer LE motor	†	Optional	Optional
Fugl-Meyer UE sensory	†	Optional	Optional
Fugl-Meyer LE sensory	†	Optional	Optional
ARAT	†	Optional	Preferred
Independent walking	Ability to walk 10 m Independently Yes/No	Optional	Preferred
10 m walk test	Seconds	Optional	Preferred
Cognitive status	MoCA test‡	Optional	Preferred
IQCODE	†	Optional	Optional
WAB-R	†	Optional	Preferred
Star cancellation	†	Optional	Optional
Depression	Yes/No	Optional	Preferred
HADS (alternatively PHQ-9)	†	Optional	Preferred
Recurrent stroke	Yes/No	Optional	Minimum
Day of recurrent stroke	Days from stroke onset	Optional	Preferred
Infection	Yes/No	Optional	Preferred
Seizure	Yes/No	Optional	Preferred
Fracture after accidental fall	Yes/No	Optional	Preferred
Physical activity	†	Optional	Preferred
PROMIS	†	Optional	Optional
SF-36	†	Optional	Optional
SIS	†	Optional	Optional
EQ-5D-5L	†	Optional	Optional
MRI	Yes/No	Optional	Optional
**Beyond 90 days: please see footnote**			

Time of evaluation (usually h for up to 72h after stroke onset; days thereafter) should be registered. Suggested evaluations at 3 months are shown. We recommend similar evaluations as those suggested for 3 months to be performed at 1 year and 3 years. For mRS, 6 month evaluation is also recommended, when possible. For references, please see Supplemental Table II.

* indicates treatment was ongoing last 2 days before stroke onset; **, information available from Charlson Comorbidity Index; ‡, including individual subitems; ǁ, within 24 h="minimum"; †, for additional details - please see Supplemental Table II.

Abbreviations for Supplemental Table I in alphabetical order:

ARAT	Action Research Arm Test
BP	Blood Pressure
CCS	causative classification of stroke system
CT	computed tomography
CTA	CT angiography
CTP	CT perfusion imaging
DSA	digital subtraction angiography
DWI	diffusion weighted imaging
ECASS	European Cooperative Acute Stroke Study
eTICI	expanded Thrombolysis In Cerebral Infarction
GCS	Glasgow Coma Scale
h	hours
HADS	Hospital Anxiety and Depression Scale
ICH	intracerebral hemorrhage
IQCODE	informant questionnaire on cognitive decline
IS	Ischemic stroke
LE	Lower extremity
m	meters
mL	milliliters
MoCA	Montreal Cognitive Assessment Scale
MRA	MRI angiography
MRI	magnetic resonance imaging
mRS	modified Rankin Scale
n	no
NIHSS	NIH stroke scale
PHQ-9	Patient Health Questionnaire-9
PROMIS	Patient-Reported Outcome Measurement Information System
SF-36	36-Item Short Form Survey
SIS	Stroke Impact Scale
TIA	Transient Ischemic Attack
TOAST	Trial of org 10172 in acute stroke treatment
UE	upper extremity
WAB-R	Western Aphasia Battery-Revised
y	Yes
