# Supplementary material for: International stroke genetics consortium recommendations for studies of genetics of stroke outcome and recovery
Source: Int J Stroke. 2021 Apr 26;17(3):260–8. doi: 10.1177/17474930211007288 (PMC8864333; doi:10.1177/17474930211007288)
Supplement: sj-pdf-3-wso-10.1177_17474930211007288 - Supplemental material for International stroke genetics consortium recommendations for studies of genetics of stroke outcome and recovery [file sj-pdf-3-wso-10.1177_17474930211007288.pdf]

Supplemental Table II.

Recommended detailed format for variables in prospective genetic stroke recovery studies. Version 1.0.1 (13 April 2021)

Variable name	Abbreviation	Format	Values	Comment	Corresponding CDE ID[1]
**1. Clinical details: Pre-stroke and demographics**					
**Pre-stroke functional status**					
Pre-stroke mRS	Pre_mRS	Numeric	0,1,2,3,4,5, Unknown		C13230
**Comorbidity**					
Charlson Comorbidity Index Score[2]	Comorb_Charlson_Ind	Numeric	0-37, Unknown	Total score. For consistency, score ***pre-stroke*** comorbidity only.<br>In addition to fields listed below, scores also added for age (score 0-4) and uncomplicated diabetes mellitus	
Dementia	Comorb_Charls_Dementia	String	Yes, No, Unknown	For total Charlson score: add score 1	
Congestive heart failure	Comorb_Charls_CHF	String	Yes, No, Unknown	For total Charlson score: add score 1	
Mild liver disease	Comorb_Charls_Liver_Dis_Mild	String	Yes, No, Unknown	For total Charlson score: add score 1<br>(if moderate to severe liver disease scores 3, mild liver disease scores zero)	
Peripheral vascular disease	Comorb_Charls_PVD	String	Yes, No, Unknown	For total Charlson score: add score 1	
Previous myocardial infarction	Comorb_Charls_MI	String	Yes, No, Unknown	For total Charlson score: add score 1	
Chronic pulmonary disease	Comorb_Charls_Pulm_Dis	String	Yes, No, Unknown	For total Charlson score: add score 1	
Connective tissue disease	Comorb_Charls_Conn_Tiss_Dis	String	Yes, No, Unknown	For total Charlson score: add score 1	
Peptic ulcer disease	Comorb_Charls_Ulcer_Dis	String	Yes, No, Unknown	For total<br>Charlson score: add score 1	
Moderate to severe renal disease	Comorb_Charls_Renal	String	Yes, No, Unknown	For total Charlson score: add score 2	
Hemiplegia	Comorb_Charls_Hemiplegia	String	Yes, No, Unknown	For total Charlson score: add score 2	
Diabetic end organ damage	Comorb_Charls_DM_end_organ	String	Yes, No, Unknown	For total Charlson score: add score 2	
Any tumor	Comorb_Charls_tumour	String	Yes, No, Unknown	For total Charlson score: add score 2<br>(if metastatic tumor scores 6, "Any tumor" scores zero)	
Leukemia	Comorb_Charls_Leukemia	String	Yes, No, Unknown	For total Charlson score: add score 2	
Lymphoma	Comorb_Charsl_Lymphoma	String	Yes, No, Unknown	For total Charlson score: add score 2	
Moderate to severe liver disease	Comorb_Charls_Liver_Dis_Sev	String	Yes, No, Unknown	For total Charlson score: add score 3	

Metastatic solid tumor	Comorb_Charls_Metast_Tum	String	Yes, No, Unknown	For total Charlson score: add score 6	
AIDS	Comorb_Charls_AIDS	String	Yes, No, Unknown	For total Charlson score: add score 6	
**Social support and living situation**					
Social support pre-stroke	Living_with_someone_pre-stroke	String	Yes, No, Unknown		C00207[a]<br>C00214[b]
Living situation pre-stroke	Housing_type	String	Apartment, Single family house, Homeless, Shelter, Dormitory, Multifamily house, Residential care, Nursing home, Unknown	Type of housing. Categories taken from CDE Supplemental Version 1.00 with addition of residential and nursing care	C55466
Residential area pre-stroke	Residential_area	String	Urban, Rural, Unknown	Self-reported	C21118
**Educational status**					
Years of education[3]	Edu_Years	Numeric	nnn (years)	Years of schooling completed	C00015[c]
**Demographics**					
Age	Age	Numeric	nnn (years)	Age at time of stroke	C0008
Sex	Sex	String	Male, Female, Other, Unknown.	Self reported	C00035
Race	Race	String	Native American; Asian; African, Black or African-American; Native Hawaiian or Other Pacific Islander; White; Other; Unknown	CDE "Race USA category" used as basis for categories, with "African" included in "Black or African American" plus "other"	C00020[d]<br>C00030[e]
Physical activity	Phys_activity_baseline	Numeric	nn	Average number of sedentary hours per day. Consider using SIMPAQ[4] to structure questions about physical activity.	
Pre-stroke cognitive decline (informant questionnaire, IQCODE)[5]	IQCODE	Numeric	n.nn	Long (26-item) IQCODE. Divide by 26. The result is a score that ranges from 1 to 5. Not available in all languages.	C13213<br>C13214<br>C13215<br>C13216<br>C13217<br>C13218<br>C13219<br>C13220<br>C13221<br>C13222<br>C13223<br>C13224

					C13225<br>C13226<br>C13227<br>C13228<br>C13229
Handedness	Dominant hand	String	Left, Right, Left and Right, Unknown	Self-reported	
**Medication**					
Medication	Med_prestroke_all	String		Comma-separated list of full drug names of all pre-stroke medications. Include only medication ongoing for the last 2 days before stroke onset	C02014
Pre-stroke ongoing anti-platelet medication	Med_prestroke_anti-plat	String	Yes, No, Unknown	Include only medication ongoing for the last 2 days before stroke onset	
	Med_prestroke_anti-plat_names	String		Comma-separated list of full drug names	C54958
Pre-stroke ongoing anticoagulant medication	Med_prestroke_anticoag	String	Yes, No, Unknown	Include only medication ongoing for the last 2 days before stroke onset	
	Med_prestroke_anticoag_names	String		Comma-separated list of full drug names	C54980
Pre-stroke anti-depressant medication	Med_prestroke_ADep	String	Yes, No, Unknown	Include only medication ongoing for the last 2 days before stroke onset	
	Med_prestroke_ADep_names	String		Comma-separated list of full drug names	
**Cardiovascular risk factors**					
Hypertension	RF_Hypertension	String	Yes, No, Unknown		C05454
Atrial fibrillation	RF_AFib	String	Yes, No, Unknown		C17466
Coronary heart disease	RF_CHD	String	Yes, No, Unknown		C21869
Diabetes mellitus	RF_DM	String	Yes, No, Unknown	For total Charlson score: add score 1<br>(if diabetic end organ damage scores 2, diabetes mellitus scores 0)	C19806
Smoking	RF_Tobacco_Smoking	String	Current, Former, Never, Unknown	Former=prior to the past 12 months	C00710[f]<br>C00711[g]
Hypercholesterolemia	RF_Hyperchol	String	Yes, No, Unknown		
Previous stroke	RF_Stroke	String	Yes, No, Unknown	For total Charlson score: add score 1	C53059
Type of previous stroke	RF_Stroke_Type	String	IS, ICH, Unknown		
Previous TIA	RF_TIA	String	Yes, No, Unknown	For total Charlson score: add score 1 if previous stroke not scoring	C13335
**2. Stroke and imaging details up to 7 days after stroke onset**					
**Stroke clinical details**					
Main stroke type	Stroke_type_main	String	IS, ICH, other	Final diagnosis.<br>All should be ischemic stroke	

Ischemic stroke subtype according to TOAST or CCS	Subtype_TOAST_CCS	String	LA, CE, SVD, OE, UND, Unknown	LA=Large artery atherosclerosis (CCS supra-aortic)<br>CE=Cardioembolism (CCS cardio-aortic)<br>SVD=Small-vessel occlusion (CCS small artery)<br>OE=Other determined etiology<br>UND=Undetermined etiology	C14225 (TOAST)<br>C14226 (CCS)
	Subtype_other_determined_etiology	String		Detail of other determined etiology (TOAST/CCS)	
Systolic blood pressure at hospital presentation	BP_sys_init	Numeric		First recorded systolic blood pressure in mmHg	C01565
Diastolic blood pressure at hospital presentation	BP_dia_init	Numeric		First recorded diastolic blood pressure in mmHg	C01507
Glucose at hospital presentation	Glu_admission	Numeric		Lab measurement (not capillary glucose), mmol/L<br>*(1 mg/dl = 0.0555 mmol/L)*	
White blood cell count initial	WBC_admission	Numeric		First white blood cell count measurement in $x10^9$ cells/L	
Blood platelet count initial	Plat_admission	Numeric		First blood platelet count $x10^9$ cells/L	
**Complications and mortality**					
Infection during first 7 days post-stroke	Infection_treated_d7orDis	String	Yes, No, Unknown	Infection treated with anti-microbials within first 7 days after stroke (or during hospital admission if discharged within 7 days)	
Survival to discharge	Survive_to_disch	String	Yes, No	Survival to discharge from hospital	
Days from stroke onset to death	Death_by_disch_days	Numeric	nn	Hours if within 72 hours	
**Stroke imaging details**					
**Before 24 hours**					
Initial imaging modality	Imag_init_modality	String	CT, MR, both, none	First imaging modality (within 24 h)	C13470
Time to initial imaging	Imag_init_time	Numeric	nn.n	Time from symptom onset to first imaging (hours, round to nearest half hour). This should be the same as NCCT time or MR time.	
CT					
Non-contrast CT performed on admission	CT_adm_NCCT_done	String	Yes, No, Unknown	Non-contrast CT	C13469
Initial CT angiography performed	CT_adm_CTA_done	String	Yes, No, Unknown		C13469
Initial CT perfusion performed	CT_adm_CTP_done	String	Yes, No, Unknown		C13469
Admission non-contrast CT time	CT_adm_NCCT_time	Numeric	nn.n	Time from symptom onset to CT (hours, round to nearest half hour)	
MRI					
MRI performed on admission	MRI_adm_done	String	Yes, No, Unknown		C13469

MRI sequences performed on admission	MRI_adm_sequences	String	T1, T2, FLAIR, SWI, GRE-T2*, DWI, ADC, CEMRA, ToF-MRA, MRPerf	Comma-separated list of sequences	C19646
Admission MRI time	MRI_adm_time	Numeric	nn.n	Time from symptom onset to start of MRI (hours, round to nearest half hour)	
General Neuroimaging					
Lesion location on imaging	Imag_init_lesion_location	String	Right-hemisphere, Left-hemisphere, Brainstem, Cerebellum, Multiple-single, Multiple-multiple, Unknown	Lesion location (presenting stroke)<br>Multiple-single = multiple infarcts, single location (of those listed). Multiple-multiple = multiple infarcts, multiple locations. If both CT and MR performed, use MR in preference.	C13766 (Site)<br>C13762 (single vascular territory)
Lesion volume on imaging	Imag_init_lesion_vol	Numeric	nnn	Lesion volume (presenting stroke) on initial imaging. If both CT and MR performed, use MR in preference.<br>If >1 ***acute*** lesion, use total lesion volume.	C13860 (MRI)<br>C14018 (CTP)
Arterial stenosis on DSA/CTA/MRA in relevant vessel	Imag_init_stenosis	String	Yes, No, Unknown	Includes intra-cranial and extra-cranial stenosis if acute stroke is considered to have occurred in the corresponding vascular territory	C13879 (modality)<br>C18892 (% stenosis)<br>C13910 (plaque severity grade)
Collateral blood flow on admission DSA/CTA/MRA	Imag_init_collat	Numeric	0,1,2,3,4,5	Collateral score[6]	C13898 (grade)<br>C13879 (modality)
**From 24 hours**					
CT					
CT 24 h post-recanalization treatment	Imaging_24h_NCCT_done	String	Yes, No, Unknown		
CT angiography 24 h post-recanalization treatment	Imaging_24h_ CTA_done	String	Yes, No, Unknown		
Time of CT 24 h post-recanalization treatment	Imaging_24h_NCCT_time	Numeric	nn	Time from recanalization treatment (intra-arterial device deployment or thrombolysis bolus if no intra-arterial treatment) to 24-h imaging (hours)	
MR					
MRI 24 h	Imaging_24h_MR_done	String	Yes, No, Unknown	MRI performed at 24 h (or within 3 days at the latest)	
Time to MR 24 h scan	Imaging_24h_MRI_time	Numeric	nn.n	Time from symptom onset to start of MRI (hours, round to nearest half hour)	
Type of MRI sequences	Imaging_24h_MRI_sequences	String	T1, T2, FLAIR, SWI, GRE-T2*, DWI, ADC, CEMRA, ToF-MRA, MRPerf	Comma-separated list of sequences	
Lesion location on MRI imaging	Imaging_24h_MRI_lesion_location	String	Right-hemisphere, Left-hemisphere, Brainstem, Cerebellum, Multiple-single, Multiple-multiple, Unknown	Lesion location (presenting stroke)<br>Multiple-single = multiple infarcts, single location (of those listed). Multiple-multiple = multiple infarcts, multiple locations. If both CT and MR performed, use MR in preference	C13766 (Site)<br>C13762 (single vascular territory)

Lesion volume on MRI imaging	Imaging_24h_MRI_lesion_vol	Numeric	nnn	Lesion volume (presenting stroke) on initial imaging. If both CT and MR performed, use MR in preference.<br>If >1 ***acute*** lesion, use total lesion volume	C13860 (MRI)<br>C14018 (CTP)
MRI microhemorrhages	Imaging_24h_MRI_microhemorrhage_count	Numeric	nn	Counted/estimated. Leave blank if blood-sensitive sequence (GRE-T2*/SWI) not performed. Use initial MR if this has blood-sensitive sequence but 24 hr MR does not	
General Neuroimaging					
Prior infarct on imaging	Imag_init_prior_infarct	String	Yes, No, Unknown	Indicates presence of chronic infarct(s) on imaging. If both CT and MR performed, use MR in preference.	C13786
Prior infarct modality	Imag_init_prior_infarct_modality	String	CT, MR, both, none		
Leukoaraiosis on imaging	Imag_init_leukoaraiosis	String	Yes, No, Unknown	Indicates presence of leukoaraiosis on imaging. If both CT and MR performed, use MR in preference.	C13810
Leuoaraiosis modality	Imag_init_leukoaraiosis_modality	String	CT, MR, both, none		
Hemorrhagic transformation	Imaging_24h_HT	String	HI1, HI2, PH1, PH2, Unknown	ECASS definitions[7]	C13843
Hemorrhagic transformation modality	Imaging_24h_HT_modality	String	CT, MR, both, none		
Edema on imaging	Cerebr_edema_CED_score_72h	Numeric	0,1,2,3	Maximum extent of edema on imaging within first 72h of stroke symptom onset, as CED score[8].<br>CED grade 0, no infarct visible; 1, focal swelling up to 1/3 of cerebral hemisphere; 2, focal swelling of >1/3 of cerebral hemisphere; 3, swelling with midline shift	C13845[k]<br>C13846[l]<br>C13847[m]
**3. Acute stroke treatment up to 7 days from stroke onset**					
**Thrombolysis**					
Treated with thrombolysis	Treat_TL	String	Yes, No, Unknown		C14396
Agent used	Treat_TL_agent	String	tPA, TNK, Other, Unknown	Alteplase, tenecteplase, other (state type)	
Time given	Treat_TL_time_min	Numeric	nnn	Time in minutes from symptom awareness to thrombolysis bolus delivery (min)	C14397
**Thrombectomy**					
Mechanical thrombectomy treatment performed	Treat_thrombect	String	Yes, No, Unknown	Yes if proceeded to groin puncture	C14401
Time to thrombectomy	Treat_thrombect_punct_time_min	Numeric	nnn	Time in minutes from symptom onset to groin puncture	C14403[h]
Cerebral perfusion post-procedure	Treat_thrombect_eTICI_post	String	0, 1, 2a, 2b, 2c, 3, Unknown	eTICI[9] (expanded TICI) score for target occlusion (vascular territory of occluded vessel causing presenting stroke)	C13891[i]
**Decompressive craniectomy**					
Treated with decompressive craniectomy	Treat_craniect	String	Yes, No, Unknown		
	Treat_craniect_type	String	Ant, Post, Unknown	Anterior or posterior fossa decompression	

**Endarterectomy/stenting**					
Treated for cervical vessel stenosis	Treat_vessel_stenosis	String	Endarterectomy, Stent, None	Treatment for stenosis of relevant (symptomatic) vessel	
**Other**					
Blood pressure treatment (acute)	Treat_BP_acute	String	Yes, No, Unknown	Acute intravenous blood pressure treatment	
**4. Functional scores up to day 7 from stroke onset**					
**Up to 24 hours**					
Initial stroke severity: NIHSS within 6 h after hospital presentation (when possible) or just before recanalization therapy	NIHSS_within_6hr_total	Numeric	0-42	NIHSS within 6 h (when possible) or before recanalization therapy	C13245
Time from stroke onset to initial NIHSS	NIHSS_within_6hr_time_Hrs	Numeric	nn.n	Hours from stroke symptom awareness to first recorded NIHSS score (round to nearest half hour). ***Preferred timing for initial NIHSS is both within 6 h and immediately before recanalization therapy***	C14191h
NIHSS subitems	NIHSS_within_6hr_item1a	Numeric	0,1,2,3	Conscious level	C13232
	NIHSS_within_6hr_item1b	Numeric	0,1,2	LOC questions	C13233
	NIHSS_within_6hr_item1c	Numeric	0,1,2	LOC commands	C13234
	NIHSS_within_6hr_item2	Numeric	0,1,2	Best gaze	C13235
	NIHSS_within_6hr_item3	Numeric	0,1,2,3	Visual	C13236
	NIHSS_within_6hr_item4	Numeric	0,1,2,3	Facial palsy	C13237
	NIHSS_within_6hr_item5a	Numeric	0,1,2,3,4	Right arm. Leave blank if amputation or joint fusion	C13238
	NIHSS_within_6hr_item5b	Numeric	0,1,2,3,4	Left arm. Leave blank if amputation or joint fusion	C13238
	NIHSS_within_6hr_item6a	Numeric	0,1,2,3,4	Right leg. Leave blank if amputation or joint fusion	C13239
	NIHSS_within_6hr_item6b	Numeric	0,1,2,3,4	Left leg. Leave blank if amputation or joint fusion	C13239
	NIHSS_within_6hr_item7	Numeric	0,1,2	Ataxia. Leave blank if amputation or joint fusion	C13240
	NIHSS_within_6hr_item8	Numeric	0,1,2		C13241
	NIHSS_within_6hr_item9	Numeric	0,1,2,3		C13242
	NIHSS_within_6hr_item10	Numeric	0,1,2	Dysarthria. Leave blank if intubated or other barrier	C13243
Physical barrier to speech at initial assessment	Speech_barrier_init_NIHSS_GCS	String	Yes, No, Unknown	Physical barrier to speech (e.g. intubation) to inform NIHSS dysarthria and GCS verbal score	
GCS subitems	GCS_initial_eyes	Numeric	1,2,3,4		C01016
	GCS_initial_verbal	Numeric/string	1,2,3,4,5	If physical barrier to speech, enter 1; refer also to "Physical barrier to speech"	
	GCS_initial_motor	Numeric	1,2,3,4,5,6		
**24-72 hours**					
NIHSS 24 h after	NIHSS_24hr_total	Numeric	0-42	NIHSS at 24 h or 24 h after recanalization therapy	C13245

recanalization therapy / 24 h after baseline NIHSS, if no recanalization therapy					
Time from stroke onset to 24-h NIHSS	NIHSS_24hr_time_hrs	Numeric	nn	Time in hours from initial NIHSS (variable NIHSS_within_6hr_time_Hrs) to “24 hr” NIHSS Preferred timing is 24 h after recanalization therapy	
NIHSS subitems	NIHSS_24h_item1a	Numeric	0,1,2,3		C13232
	NIHSS_24hr_item1b	Numeric	0,1,2		C13233
	NIHSS_24hr_item1c	Numeric	0,1,2		C13234
	NIHSS_24hr_item2	Numeric	0,1,2		C13235
	NIHSS_24hr_item3	Numeric	0,1,2,3		C13236
	NIHSS_24hr_item4	Numeric	0,1,2,3		C13237
	NIHSS_24hr_item5a	Numeric	0,1,2,3,4	Right arm	C13238
	NIHSS_24hr_item5b	Numeric	0,1,2,3,4	Left arm	C13238
	NIHSS_24hr_item6a	Numeric	0,1,2,3,4	Right leg	C13239
	NIHSS_24hr_item6b	Numeric	0,1,2,3,4	Left leg	C13239
	NIHSS_24hr_item7	Numeric	0,1,2		C13240
	NIHSS_24hr_item8	Numeric	0,1,2		C13241
	NIHSS_24hr_item9	Numeric	0,1,2,3		C13242
	NIHSS_24hr_item10	Numeric	0,1,2		C13243
	NIHSS_24hr_item11	Numeric	0,1,2		C13244
Physical barrier to speech at 24 h assessment	Speech_barrier_24h_NIHS	String	Yes, No, Unknown	Physical barrier to speech (e.g. intubation) to inform NIHSS dysarthria score	
SAFE score within 72 hours	SAFE_score	Numeric	0,1,2,3,4,5,6,7,8,9,10	Shoulder abduction finger extension score within first 72 h (sum of MRC grades (0-5) for shoulder abduction and finger extension)	
**7 days or discharge if earlier**					
NIHSS at day 7 or discharge if earlier, score of measurement	NIHSS_d7orDis_total	Numeric	0-42		C13245
NIHSS at day 7 or discharge if earlier, time of measurement	NIHSS_d7orDis_time_days	Numeric	n	Full days from symptom onset to recorded NIHSS	
NIHSS subitems	NIHSS_d7orDis_item1a	Numeric	0,1,2,3		C13232
	NIHSS_d7orDis_item1b	Numeric	0,1,2		C13233
	NIHSS_d7orDis_item1c	Numeric	0,1,2		C13234
	NIHSS_d7orDis_item2	Numeric	0,1,2		C13235
	NIHSS_d7orDis_item3	Numeric	0,1,2,3		C13236
	NIHSS_d7orDis_item4	Numeric	0,1,2,3		C13237
	NIHSS_d7orDis_item5a	Numeric	0,1,2,3,4		C13238
	NIHSS_d7orDis_item5b	Numeric	0,1,2,3,4		C13238
	NIHSS_d7orDis_item6a	Numeric	0,1,2,3,4		C13239

	NIHSS_d7orDis_item6b	Numeric	0,1,2,3,4		C13239
	NIHSS_d7orDis_item7	Numeric	0,1,2		C13240
	NIHSS_d7orDis_item8	Numeric	0,1,2		C13241
	NIHSS_d7orDis_item9	Numeric	0,1,2,3		C13242
	NIHSS_d7orDis_item10	Numeric	0,1,2		C13243
	NIHSS_d7orDis_item11	Numeric	0,1,2		C13244
Physical barrier to speech at day 7/discharge assessment	Speech_barrier_24h_NIHS	String	Yes, No, Unknown	Physical barrier to speech (e.g. intubation) to inform NIHSS dysarthria score and cognitive testing	
**Sensorimotor function**					
Fugl-Meyer UE motor day 7/discharge	FM_UE_M_d7orDis_total	Numeric	0-66	Total upper extremity score. For more detailed breakdown of scoring, see[10]	
Fugl-Meyer UE motor subitems	FM_UE_M_d7orDis_SA	Numeric	0-36	Shoulder-arm	
	FM_UE_M_d7orDis_W	Numeric	0-10	Wrist	
	FM_UE_M_d7orDis_H	Numeric	0-14	Hand	
	FM_UE_M_d7orDis_Co	Numeric	0-6	Co-ordination/speed	
Fugl-Meyer LE motor day 7/discharge	FM_LE_M_d7orDis_total	Numeric	0-66	Total lower extremity score. For more detailed breakdown of scoring, see[10]	
Fugl-Meyer LE motor subitems	FM_LE_M_d7orDis	Numeric	0-28		
	FM_LE_M_d7orDis_Co	Numeric	0-6	Co-ordination/speed	
Fugl-Meyer Upper Extremity sensory at day 7/discharge	FM_UE_S_d7orDis	Numeric	0-12		
Fugl-Meyer Lower Extremity sensory at day 7/discharge	FM_LE_S_d7orDis	Numeric	0-12		
ARAT at day 7/discharge	ARAT_ d7orDis	Numeric	0-57	Total ARAT score[10]	
ARAT subitems	ARAT_ d7orDis_grasp	Numeric	0-18		
	ARAT_ d7orDis_grip	Numeric	0-12		
	ARAT_ d7orDis_pinch	Numeric	0-18		
	ARAT_d7_gross_movt	Numeric	0-9		
Independent walking at day 7/discharge	Walk_d7_10m_able	String	Yes, No, Unknown	Ability to walk 10 meters independently, with walking aid if necessary	
10 m walk test	Walk_d7orDis_10m_time	Numeric	nn	Time taken to walk 10 meters, if able	
**Cognitive function**					
Cognitive status (MoCA) at 7 days or earlier at discharge	MoCA_d7orDis_total	Numeric	0-30	Total score, including 1-point correction for ≤12 years of education (Max 30)	
MoCA version used at day 7 or discharge	MoCA_version_d7orDis	String		Version number and/or language. MoCA version 7.0 preferred if available	
MoCA subitems	MoCA_ d7orDis _VS_Exec	Numeric	0-5	Visuospatial/executive functions	
	MoCA_ d7orDis _Nam	Numeric	0-3	Naming	
	MoCA_ d7orDis _DSpan	Numeric	0-2	Digit span	
	MoCA_ d7orDis _Vig	Numeric	0-1	Vigilance	

	MoCA_ d7orDis _Cal	Numeric	0-3	Calculation	
	MoCA_ d7orDis _Rep	Numeric	0-2	Repetition	
	MoCA_ d7orDis _VF	Numeric	0-1	Verbal fluency	
	MoCA_ d7orDis _Abs	Numeric	0-2	Abstraction	
	MoCA_ d7orDis _DRecall	Numeric	0-5	Delayed recall	
	MoCA_ d7orDis _Orient	Numeric	0-6	Orientation	
	MoCA_d7orDis_Edu	Numeric	0-1	Education <12 years =1, else 0	
**5. Considerations and treatment information at 90 days/3 months and beyond**					
Social support at day 90 post-stroke	Living_with_someone_d90	String	Yes, No, Unknown		C00207[a] C00214[b]
Rehab treatment	Rehab_treatment	String	Yes, No, Unknown	Formal rehab services received following hospital discharge.	
Rehab start day	Rehab_start_day	Number	n, NA	Rehab start day after stroke onset	
Rehab received to 90 days	Rehab_d90_weeks	Number	nn	Number of weeks therapy received. This includes all rehab received since stroke onset, including acute hospital stay	C05132 (frequency) C05133 (duration)
	Rehab_d90_hours_per_week	Number	nn	Average number of hours of rehab received per week (all therapies combined), for weeks recorded above	
Other rehab adjunct	Rehab_adjunct_d90	String	[name of adjunct], none	Name any relevant rehab adjunct(s) used	
Antidepression treatment on day 90	Med_d90_ADep	String	Yes, No, Unknown		
	Med_d90_ADep_names	String		Comma-separated list of full drug names	
Antiplatelet treatment on day 90	Med_d90_antiplatelet	String	Yes, No, Unknown		
	Med_d90_antiplatelet_names	String		Comma-separated list of full drug names	
Anticoagulant treatment on day 90	Med_d90_anticoag	String	Yes, No, Unknown		
	Med_d90_anticoag_names	String		Comma-separated list of full drug names	
Dyslipidemia treatment on day 90	Med_d90_lipid	String	Yes, No, Unknown		
	Med_d90_lipid_names	String		Comma-separated list of full drug names	
Blood pressure treatment on day 90	Med_d90_blood_pressure	String	Yes, No, Unknown		
	Med_d90_blood_pressure_names	String		Comma-separated list of full drug names	
**6. Evaluation at 90 days/3 months and beyond**					
At 90-days					
90-day assessment, days since stroke	d90_assess_days	Numeric	nn	Full days from symptom awareness to 90-day assessment. All 90-day assessments and outcomes assumed to have taken place on this day	
**Global function, independence, disability**					
NIHSS at 90 days	NIHSS_d90_total	Numeric	0-42		C13245
NIHSS subitems	NIHSS_d90_item1a	Numeric	0,1,2,3		C13232
	NIHSS_ d90 _item1b	Numeric	0,1,2		C13233
	NIHSS_ d90 _item1c	Numeric	0,1,2		C13234
	NIHSS_ d90 _item2	Numeric	0,1,2		C13235

	NIHSS_ d90 _item3	Numeric	0,1,2,3		C13236
	NIHSS_ d90 _item4	Numeric	0,1,2,3		C13237
	NIHSS_ d90 _item5a	Numeric	0,1,2,3,4	Right arm	C13238
	NIHSS_ d90 _item5b	Numeric	0,1,2,3,4	Left arm	C13238
	NIHSS_ d90 _item6a	Numeric	0,1,2,3,4	Right leg	C13239
	NIHSS_ d90 _item6b	Numeric	0,1,2,3,4	Left leg	C13239
	NIHSS_ d90 _item7	Numeric	0,1,2		C13240
	NIHSS_ d90 _item8	Numeric	0,1,2		C13241
	NIHSS_ d90 _item9	Numeric	0,1,2,3		C13242
	NIHSS_ d90 _item10	Numeric	0,1,2		C13243
	NIHSS_ d90 _item11	Numeric	0,1,2		C13244
mRS at 90 days	mRS_d90	Numeric	0,1,2,3,4,5,6, Unknown		C13230
Barthel Index	Barthel_d90_total	Numeric	0-100	Total Barthel Index score[11]	
Barthel subitems	Feeding	Numeric	0,5,10		
	Transfers to chair	Numeric	0,5,10,15		
	Personal toilet	Numeric	0,5		
	Getting on and off toilet	Numeric	0,10		
	Bathing	Numeric	0,5		
	Walking/wheelchair	Numeric	0,5,10,15		
	Stairs	Numeric	0,5,10		
	Dressing	Numeric	0,5,10		
	Bowel continence	Numeric	0,5,10		
	Bladder control	Numeric	0,5,10		
**Sensorimotor function**					
Fugl-Meyer UE motor at day 90 total	FM_UE_M_d90_total	Numeric	0-66	Total score	
Fugl-Meyer UE motor subitems	FM_UE_M_d90_SA	Numeric	0-36	Shoulder-arm	
	FM_UE_M_d90_W	Numeric	0-10	Wrist	
	FM_UE_M_d90_H	Numeric	0-14	Hand	
	FM_UE_M_d90_Co	Numeric	0-6	Upper limb co-ordination/speed	
Fugl-Meyer LE motor at day 90 total	FM_LE_M_d90_total	Numeric	0-34	Total lower extremity score. For more detailed breakdown of scoring, see[10]	
Fugl-Meyer LE motor subitems	FM_LE_M_d90	Numeric	0-28		
	FM_LE_M_d90_Co	Numeric	0-6	Co-ordination/speed	
Fugl-Meyer Upper Extremity sensory at day 90	FM_UE_S_d90	Numeric	0-12		
Fugl-Meyer Lower Extremity sensory at day 90	FM_LE_S_d90	Numeric	0-12		
ARAT at day 90	ARAT_d90	Numeric	0-57	Total ARAT score[10]	

ARAT subitems	ARAT_d90_grasp	Numeric	0-18		
	ARAT_d90_grip	Numeric	0-12		
	ARAT_d90_pinch	Numeric	0-18		
	ARAT_d90_gross_movt	Numeric	0-9		
Independent walking at day 90	Walk_d90_10m_able	String	Yes, No, Unknown	Ability to walk 10 meters independently, with walking aid if necessary	
10 m walk test	Walk_d90_10m_time	Numeric	nn	Time taken to walk 10 meters, if able	
**Cognitive function, language function and depression**					
Cognitive status (MoCA) at day 90	MoCA_d7orDis_total	Numeric	0-30	Total score, including 1 point correction for ≤12 years of education.	
MoCA version used at day 90	MoCA_version_d90	String		Version number and/or language. Version 7.0 preferred if available.	
MoCA subitems	MoCA_d90_VS_Exec	Numeric	0-5	Visuospatial/executive functions	
	MoCA_d90_Nam	Numeric	0-3	Naming	
	MoCA_d90_DSpan	Numeric	0-2	Digit span	
	MoCA_d90_Vig	Numeric	0-1	Vigilance	
	MoCA_d90_Cal	Numeric	0-3	Calculation	
	MoCA_d90_Rep	Numeric	0-2	Repetition	
	MoCA_d90_VF	Numeric	0-1	Verbal fluency	
	MoCA_d90_Abs	Numeric	0-2	Abstraction	
	MoCA_d90_DRecall	Numeric	0-5	Delayed recall	
	MoCA_d90_Orient	Numeric	0-6	Orientation	
Post-stroke cognitive decline (IQCODE)	IQCODE_d90	Numeric	n	IQCODE score for ***post-stroke period only***, to detect post-stroke cognitive decline. Ask questions about cognitive change "since before their stroke"	C13213<br>C13214<br>C13215<br>C13216<br>C13217<br>C13218<br>C13219<br>C13220<br>C13221<br>C13222<br>C13223<br>C13224<br>C13225<br>C13226<br>C13227<br>C13228<br>C13229
Western Aphasia Battery – Revised (WAB-R)[12]	WAB-R_d90	Numeric	nn	WAB-R total score	

Star cancellation[13]	Star_cancel_omissions_d90	Numeric	nn	Number of omissions	
Depression at day 90	Depr_d90	String	Yes, No, Unknown	Depression diagnosed or treated during or after hospital admission up to day 90	
Depression scale score at day 90	Depr_d90_score	Numeric	nn		
Depression measure used to generate score at day 90	Depr_d90_measure	String	HADS, PHQ9, Other, Unknown		
**Complications and mortality**					
Recurrent stroke day 2-90	Recur_Str_d2-90	String	Yes, No, Unknown	Day 2 to 90 inclusive	
	Recur_Str_d2-90_source	String	Patient, Records, Unknown	Patient reported or derived from medical records	
Days from onset to recurrent stroke	Recur_Str_by_d90_days	Numeric	nn		
Infection from day 7 or discharge to day 90	Infect_treat_d7orDis-d90	String	Yes, No, Unknown	Any infection treated between day 7 or hospital discharge and day 90	
Seizure	Seizure_d7orDis-d90	String	Yes, No, Unknown	Any seizure treated between day 7 or hospital discharge and day 90 OR ongoing seizure treatment	C05460
Fracture after accidental fall	Fracture_fall_d90	String	Yes, No, Unknown	Any fracture from accidental fall ***from symptom awareness to day 90***	
Survival to day 90	Survive_to_d90	String	Yes, No	Survival to discharge from hospital	
Days from onset to death	Death_by_d90_days	Numeric	nn		
**Physical activity**					
Physical activity	Phys_activity_d90	Numeric	nn	Average number of sedentary hours per day. Consider using SIMPAQ[4] to structure questions about physical activity	
**Patient-reported outcome measures (PROMs)**					
PROMIS summary scores[14]	PROMIS_d90_Summ_Ment_health	Numeric	nn	PROMIS Mental Component T score (Mean 50, SD 10)	C20687
	PROMIS_d90_Summ_Phys_health	Numeric	nn	PROMIS Physical Component T score (Mean 50, SD 10)	C20687
PROMIS global health subitems	PROMIS_d90_GH_gen_health	Numeric	1-5	General health	
	PROMIS_d90_GH_QoL	Numeric	1-5	Quality of life	C54688
	PROMIS_d90_GH_phys_health	Numeric	1-5	Physical health	C54689
	PROMIS_d90_GH_ment_health	Numeric	1-5	Mental health	C54690
	PROMIS_d90_GH_social satisfact	Numeric	1-5	Social satisfaction	C54691
	PROMIS_d90_GH_phys_activ	Numeric	1-5	Physical activities	C54693
	PROMIS_d90_GH_pain	Numeric	1-5	Pain. Scored as 0-10 but re-scored to 1-5	
	PROMIS_d90_GH_fatigue	Numeric	1-5	Fatigue	C54695
	PROMIS_d90_GH_social_activ	Numeric	1-5	Social activities	C54692
	PROMIS_d90_GH_emot_probs	Numeric	1-5	Emotional problems	C54694
SF-36 component scores[15]	SF36_d90_Ment_health	Numeric		36-item short form survey mental health component score	

	SF36_d90_Phys_health	Numeric		36-item short form survey physical health component score	
SF-36 subitems	SF36_d90_phys_func	Numeric		SF36 physical function item	
	SF36_d90_role_phys	Numeric		SF36 role function (physical) item	
	SF36_d90_bodily_pain	Numeric		SF36 bodily pain item	C08521
	SF36_d90_general_health	Numeric		SF36 general health item	C08500
	SF36_d90_vitality	Numeric		SF36 vitality item	
	SF36_d90_social_func	Numeric		SF36 social functioning item	
	SF36_d90_role_emot	Numeric		SF36 role function (emotional) item	
	SF36_d90_mental_health	Numeric		SF36 mental health item	
Stroke Impact Scale (SIS)[16] domains	SIS_d90_strength	Numeric	0-100	Stroke Impact Scale v3.0 strength domain	
	SIS_d90_hand_func	Numeric	0-100	SIS v3.0 hand function domain	
	SIS_d90_ADL	Numeric	0-100	SIS v3.0 activities of daily living domain	
	SIS_d90_mobility	Numeric	0-100	SIS v3.0 mobility domain	
	SIS_d90_communication	Numeric	0-100	SIS v3.0 communication domain	
	SIS_d90_emotion	Numeric	0-100	SIS v3.0 emotion domain	
	SIS_d90_mem_think	Numeric	0-100	SIS v3.0 memory and thinking domain	
	SIS_d90_particip_role	Numeric	0-100	SIS v3.0 participation and role function	
	SIS_d90_recovery	Numeric	0-100	SIS v3.0 self-assessed recovery	
EQ-5D-5L[17]	EQ-5D-5L_d90_num	Numeric	nnnnn	Each digit is the level (1-5) of one of the five EQ-5D dimensions: mobility, self-care, usual activities, pain/discomfort and anxiety/depression), resulting in a 5-digit "Health State"	
EQ-5D-5L VAS[17]	EQ-5D-5L_d90_VAS	Numeric	0-100	Health today on the EQ-5D-5L visual analogue scale	
**Imaging**					
MRI performed at day 90	MRI_d90_done	String	Yes, No, Unknown		
MRI performed at day 90, days post-stroke	MRI_d90_days	Numeric	nnn	Full days from symptom awareness to MRI scan	
**Beyond 90 days: please see footnote**					

The Table shows suggested evaluations at 90 days. We recommend similar evaluations at 1 year and 3 years. For mRS, 6 month evaluation is also recommended, when possible.

NINDS CDE[1] variable IDs are listed in last column. The CDE variables do not always fully align with the suggested variables and this is reflected in the added comments below.

[a] CDE records marital/partnered status: [Never married; Married; Domestic partnership; Divorced; Separated; Widowed].

[b] CDE records number of people participant is living or cohabiting with.

[c] CDE records highest grade or school or highest degree achieved.

[d,e] CDE separates race[d] [American Indian or Alaska Native; Asian; Black or African-American; Native Hawaiian or Other Pacific Islander; White; Unknown; Not reported] from ethnicity[e] [Hispanic or Latino; Not Hispanic or Latino; Unknown; Not reported; Other, specify].

[f,g] CDE separates current tobacco use[f] from regular use prior to the past 12 months[g].

[h] In CDE, measures are coupled to specific dates/times rather than relative to symptom onset.

[i] CDE excludes TICI 2c.

[j] CDE includes hemorrhagic transformation radiologic types.

[k] CDE records presence of any shift [Yes; No; Unknown].

[l] CDE records type of shift [midline shift; septal shift; pineal shift].

[m] CDE records shift measurement (millimeters).

Abbreviations for Supplemental Table II in alphabetical order:

ADC	Apparent diffusion coefficient
ARAT	Action Research Arm Test
BP	Blood Pressure
CCS	causative classification of stroke system
CDE	CDE, Common Data Elements (NINDS)
CEMRA	Contrast enhanced MR angiography
CT	computed tomography
CTA	CT angiography
CTP	CT perfusion imaging
DSA	digital subtraction angiography
DWI	diffusion weighted imaging
ECASS	European Cooperative Acute Stroke Study
eTICI	expanded Thrombolysis In Cerebral Infarction score
FLAIR	Fluid-attenuated inversion recovery
GCS	Glasgow Coma Scale
GRE-T2*	Gradient Echo-T2*
h	hours
HADS	Hospital Anxiety and Depression Scale

HI	Hemorrhagic infarction
ICH	intracerebral hemorrhage
IQCODE	informant questionnaire on cognitive decline
IS	Ischemic stroke
LE	Lower extremity
m	meters
mL	millilitres
MoCA	Montreal Cognitive Assessment Scale
MRA	MRI angiography
MRI	magnetic resonance imaging
MRPerf	MR perfusion imaging
MRC	Medical Research Council
mRS	modified Rankin Scale
NIHSS	NIH stroke scale
OAC	oral anticoagulation
PH	Parenchymal hematoma
PHQ-9	Patient Health Questionnaire-9
PROMIS	Patient-Reported Outcome Measurement Information System
SAFE	Shoulder abduction finger extension
SF-36	36-Item Short Form Survey
SIS	Stroke Impact Scale
SWI	Susceptibility-weighted imaging
TIA	Transient Ischemic Attack
TNK	Tenecteplase
TOAST	Trial of org 10172 in acute stroke treatment
ToF-MRA	Time-of-Flight MR angiography
tPA	tissue plasminogen activator
UE	upper extremity
WAB-R	Western Aphasia Battery-Revised

References

1. Ninds common data elements. https://commondataelements.ninds.nih.gov/cde-catalog
2. Charlson ME, Pompei P, Ales KL, MacKenzie CR. A new method of classifying prognostic comorbidity in longitudinal studies: Development and validation. *J Chronic Dis*. 1987;40:373-383
3. Lee JJ, Wedow R, Okbay A, Kong E, Maghzian O, Zacher M, et al. Gene discovery and polygenic prediction from a genome-wide association study of educational attainment in 1.1 million individuals. *Nat Genet*. 2018;50:1112-1121
4. Rosenbaum S, Ward PB, International Working G. The simple physical activity questionnaire. *Lancet Psychiatry*. 2016;3:e1
5. Jorm AF. The informant questionnaire on cognitive decline in the elderly (iqcode): A review. *Int Psychogeriatr*. 2004;16:275-293
6. Maas MB, Lev MH, Ay H, Singhal AB, Greer DM, Smith WS, et al. Collateral vessels on ct angiography predict outcome in acute ischemic stroke. *Stroke*. 2009;40:3001-3005
7. Hacke W, Kaste M, Fieschi C, Toni D, Lesaffre E, von Kummer R, et al. Intravenous thrombolysis with recombinant tissue plasminogen activator for acute hemispheric stroke. The european cooperative acute stroke study (ecass). *JAMA*. 1995;274:1017-1025
8. Strbian D, Meretoja A, Putaala J, Kaste M, Tatlisumak T, Helsinki Stroke Thrombolysis Registry G. Cerebral edema in acute ischemic stroke patients treated with intravenous thrombolysis. *Int J Stroke*. 2013;8:529-534
9. Liebeskind DS, Bracard S, Guillemin F, Jahan R, Jovin TG, Majoie CB, et al. Etici reperfusion: Defining success in endovascular stroke therapy. *J Neurointerv Surg*. 2019;11:433-438
10. Kwakkel G, Lannin NA, Borschmann K, English C, Ali M, Churilov L, et al. Standardized measurement of sensorimotor recovery in stroke trials: Consensus-based core recommendations from the stroke recovery and rehabilitation roundtable. *Int J Stroke*. 2017;12:451-461
11. Mahoney FI, Barthel DW. Functional evaluation: The barthel index. *Md State Med J*. 1965;14:61-65
12. Western aphasia battery – revised. https://www.strokengine.ca/en/family/wab-see-the-measure/
13. Star cancellation test. https://www.strokengine.ca/en/family/sct_family/
14. Hays RD, Bjorner JB, Revicki DA, Spritzer KL, Cella D. Development of physical and mental health summary scores from the patient-reported outcomes measurement information system (promis) global items. *Qual Life Res*. 2009;18:873-880
15. Ware JE, Jr., Gandek B. Overview of the sf-36 health survey and the international quality of life assessment (iqola) project. *J Clin Epidemiol*. 1998;51:903-912
16. Duncan PW, Lai SM, Tyler D, Perera S, Reker DM, Studenski S. Evaluation of proxy responses to the stroke impact scale. *Stroke*. 2002;33:2593-2599
17. https://euroqol.org/
